# Use of 5-Alpha-Reductase Inhibitors Did Not Increase the Risk of Cardiovascular Diseases in Patients with Benign Prostate Hyperplasia: A Five-Year Follow-Up Study

**DOI:** 10.1371/journal.pone.0119694

**Published:** 2015-03-24

**Authors:** Teng-Fu Hsieh, Yu-Wan Yang, Shang-Sen Lee, Tien-Huang Lin, Hsin-Ho Liu, Tsung-Hsun Tsai, Chi-Cheng Chen, Yung-Sung Huang, Ching-Chih Lee

**Affiliations:** 1 Department of Urology, Taichung Tzu Chi Hospital, Buddhist Tzu Chi Medical Foundation, Taichung, Taiwan; 2 School of Medicine, Tzu Chi University, Hualian, Taiwan; 3 Department of Neurology, China Medical University Hospital, Taichung, Taiwan; 4 School of Medicine, China Medical University, Taichung, Taiwan; 5 Division of Neurology, Department of Internal Medicine, Dalin Tzu Chi Hospital, Buddhist Tzu Chi Medical Foundation, Chiayi, Taiwan; 6 Department of Otolaryngology, Dalin Tzu Chi Hospital, Buddhist Tzu Chi Medical Foundation, Chiayi, Taiwan; 7 Department of Education, Dalin Tzu Chi Hospital, Buddhist Tzu Chi Medical Foundation, Chiayi, Taiwan; 8 Center for Clinical Epidemiology and Biostatistics, Dalin Tzu Chi Hospital, Buddhist Tzu Chi Medical Foundation, Chiayi, Taiwan; National Health Research Institutes, TAIWAN

## Abstract

**Background:**

This nationwide population-based study investigated the risk of cardiovascular diseases after 5-alpha-reductase inhibitor therapy for benign prostate hyperplasia (BPH) using the National Health Insurance Research Database (NHIRD) in Taiwan.

**Methods:**

In total, 1,486 adult patients newly diagnosed with BPH and who used 5-alpha-reductase inhibitors were recruited as the study cohort, along with 9,995 subjects who did not use 5-alpha-reductase inhibitors as a comparison cohort from 2003 to 2008. Each patient was monitored for 5 years, and those who subsequently had cardiovascular diseases were identified. A Cox proportional hazards model was used to compare the risk of cardiovascular diseases between the study and comparison cohorts after adjusting for possible confounding risk factors.

**Results:**

The patients who received 5-alpha-reductase inhibitor therapy had a lower cumulative rate of cardiovascular diseases than those who did not receive 5-alpha-reductase inhibitor therapy during the 5-year follow-up period (8.4% vs. 11.2%, P=0.003). In subgroup analysis, the 5-year cardiovascular event hazard ratio (HR) was lower among the patients older than 65 years with 91 to 365 cumulative defined daily dose (cDDD) 5-alpha-reductase inhibitor use (HR=0.63, 95% confidence interval (CI) 0.42 to 0.92; P=0.018), however there was no difference among the patients with 28 to 90 and more than 365 cDDD 5-alpha-reductase inhibitor use (HR=1.14, 95% CI 0.77 to 1.68; P=0.518 and HR=0.83, 95% CI 0.57 to 1.20; P=0.310, respectively).

**Conclusions:**

5-alpha-reductase inhibitor therapy did not increase the risk of cardiovascular events in the BPH patients in 5 years of follow-up. Further mechanistic research is needed.

## Introduction

The indications for 5-alpha-reductase inhibitor therapy, including finasteride and dutasteride, in benign prostate hyperplasia (BPH) treatment have been reported to be symptomatic disease and a large prostate volume [1–3]. 5-alpha-reductase inhibitor therapy leads to near maximal suppression of dihydrotestosterone, which results in a reduction of serum androgens levels, prostate gland growth and the bothersome symptoms from BPH [2–5]. Because of the effectiveness of 5-alpha-reductase inhibitor therapy in BPH patients, the prevalence of the use of 5-alpha-reductase inhibitors for BPH has steadily increased [6]. However, several recent studies have demonstrated increased cardiovascular mortality in men with lower serum androgen levels, especially those with existing cardiovascular disease [7–9]. It has been reported that prostate cancer patients who receive androgen deprivation therapy to decrease their serum androgens level are at an increased risk of cardiovascular diseases [10–12]. Therefore, the risk of cardiovascular diseases should be taken into consider when using 5-alpha-reductase inhibitor therapy for BPH.

Many studies have evaluated the cardiovascular risk associated with 5-alpha-reductase inhibitor therapy, however no consistent evidence of a significant association between 5-alpha-reductase inhibitor therapy and the risk of cardiovascular adverse events has been found [13–16]. Hence, further studies to determine the association between 5-alpha-reductase inhibitor therapy and cardiovascular diseases are warranted.

We conducted this population-based nationwide study using the National Health Insurance Research Database (NHIRD) in Taiwan to investigate the association between 5-alpha-reductase inhibitor therapy and cardiovascular diseases. Several studies have used the NHIRD to investigate associations between different diseases [17–19]. The high accuracy of the NHIRD in recording ischemic stroke diagnoses and aspirin prescriptions has been reported, and the NHIRD appears to be a valid resource for population-based research in ischemic stroke [20]. This nationwide population-based dataset allows researchers to trace the medical service utilization history of all citizens in Taiwan, and provides a unique opportunity to compare the risk of cardiovascular diseases in patients receiving 5-alpha-reductase inhibitor therapy with that of the general population.

## Materials and Methods

### Ethics Statements

This study was initiated after being approved by the Institutional Review Board of the Buddhist Dalin Tzu Chi General Hospital, Taiwan (IRB approved protocol number is B10302009). Because the identification numbers and personal information of the individuals included in this study were not included in the secondary files, the review board stated that written consent from the patients was not required.

### Patients and Study Design

Taiwan implemented a National Health Insurance (NHI) program in 1995 to provide comprehensive health care coverage. Enrollment in this government-run, universal, single-payer insurance system is mandatory, and currently up to 99% of the 23 million residents of Taiwan receive medical care through the NHI program. In addition, over 97% of the hospitals and clinics in Taiwan are contracted to provide health care services [21], which are reimbursed by the Bureau of NHI, and all data related to these services are collected and input into the NHIRD by the National Health Research Institutes to provide a comprehensive record of medical care. The data consist of ambulatory care records, inpatient care records, and the registration files of the insured, and the database includes all claims data from the NHI program. The NHI Bureau of Taiwan randomly reviews the charts of one out of every 100 ambulatory cases, and one out of every 20 inpatient cases, as well as performing patient interviews to verify the accuracy of the diagnosis [22].

This study used the 2003 to 2008 NHIRD. Because the data consisted of de-identified secondary data released to the public for research, this study was exempt from full review by the Institutional Review Board.

The study design featured a study cohort and a comparison cohort. We selected all patients who had been newly diagnosed with BPH (International Classification of Diseases, Ninth Revision, Clinical Modification (ICD-9-CM) code 600.xx) and who were followed up between 2003 and 2005. We then excluded the patients who had been newly diagnosed with cerebrovascular disease (ICD-9-CM: 430.xx-438.xx), myocardial infarction (ICD-9-CM: 410.xx-411.xx) and coronary artery disease (ICD-9-CM: 413.xx-414.xx) before the index date.

We then selected the patients who had received 5-alpha-reductase inhibitor therapy, including finasteride and dutasteride, between 2003 and 2005 as the study cohort and used the date of initiation of 5-alpha-reductase inhibitor therapy as the patient’s index date. The control cohort included all the other patients with BPH who did not receive 5-alpha-reductase inhibitor therapy. The independent variables were comorbid disorders, geographical area of residence and urbanization level, and socioeconomic status (SES).

The defined daily dose (DDD) recommended by the WHO is a unit for measuring a prescribed amount of drug; it is the assumed average maintenance dose per day of a drug consumed for its main indication in adults. By using the following formula, we could compare any dose of 5-alpha-reductase inhibitors based on the same standard and estimate the cumulative dose as the followings:

(total amount of drug)/(amount of drug in a DDD) = number of cumulative DDDs [23].

The cumulative DDD (cDDD), which indicates the duration of exposure, was estimated as the sum of dispensed DDD of a 5-alpha-reductase inhibitor to compare their use with the risk of cardiovascular diseases. Tsan et al used this definition to study statins and the risk of hepatocellular carcinoma in patients with hepatitis B virus infection [24]. To examine the dose-effect relationship, we categorized the use of 5-alpha-reductase inhibitors into four groups in each cohort (less than 28, 28 to 90, 91 to 365, and more than 365 cDDDs) because the duration of the refill card was 3 months. The patients who used 5-alpha-reductase inhibitors for less than 28 cDDDs were defined as nonusers of 5-alpha-reductase inhibitors.

### Other Variables

The subjects were classified into three groups: (1) low SES: lower than US$528 per month (New Taiwan Dollars (NT$) 15840); (2) moderate SES: between US$528–833 per month (NT$15841–25000); and (3) high SES: US$833 per month (NT$25001) or more [25]. We selected NT$15,840 as the low income level cutoff point because this was the government-stipulated minimum wage for full-time employees in Taiwan in 2006. The geographic regions where the individuals resided were recorded as northern, central, southern, and eastern Taiwan.

### Statistical Analysis

SPSS version 15 software (SPSS Inc., Chicago, IL, USA) was used for all data analyses. Pearson’s chi-square test was used for categorical variables such as SES, geographic region of residence, and comorbidities. Continuous variables were analyzed using a one-way ANOVA test. The cumulative risk of cardiovascular diseases for those who did and did not receive 5-alpha-reductase inhibitor therapy was estimated with Kaplan-Meier survival curves. A Cox proportional hazards regression model adjusted for patient characteristics (age, co-morbidities, SES, and geographic region) was used to analyze the association of 5-alpha-reductase inhibitor usewith subsequent cardiovascular diseases during the 5-year follow-up period. We calculated hazard ratios (HRs) along with 95% confidence intervals (CIs) using a significance level of 0.05. A two-sided P-value (P<0.05) was used to determine statistical significance.

## Results

A total of 11,481 patients with BPH were included in our study cohort, of whom 1,486 received 5-alpha-reductase inhibitor therapy and 9,995 did not. The demographic characteristics and selected morbidities for the two cohorts are shown in Table 1. The patients who received 5-alpha-reductase inhibitor therapy were more likely to be older, reside in an urban area or central or eastern Taiwan, and have a lower SES than the controls. However, there was no difference in Charlson Comorbidity Index Score between the two cohorts.

**Table 1:** Demographic characteristics of the study cohort and controls (n = 11,481).

Characteristics	With 5ARI	Without 5ARI	*p*-value
Patient no.	1486	9995	
Mean age, years (±SD)	69±9.2	67±10.9	<0.001
Charlson Comorbidity Index Score			0.693
0	880(59.2)	5957(59.6)	
1	351(23.6)	2439(24.4)	
2	161(10.8)	1003(10.0)	
> = 3	94(6.3)	596(6.0)	
Socioeconomic status			<0.001
Low	925(62.2)	5570(55.7)	
Moderate	369(24.8)	2883(28.8)	
High	192(12.9)	1542(15.4)	
Urbanization			<0.001
Urban	505(34.0)	2866(28.7)	
Suburban	603(40.6)	4208(42.1)	
Rural	378(25.4)	2921(29.2)	
Geographic region			<0.001
Northern	749(50.4)	5013(50.2)	
Central	255(17.2)	1673(16.7)	
Southern	397(26.7)	3039(30.4)	
Eastern	85(5.7)	270(2.7)	

Abbreviation: 5ARI, 5-alpha reductase inhibitor.

**Fig 1:**
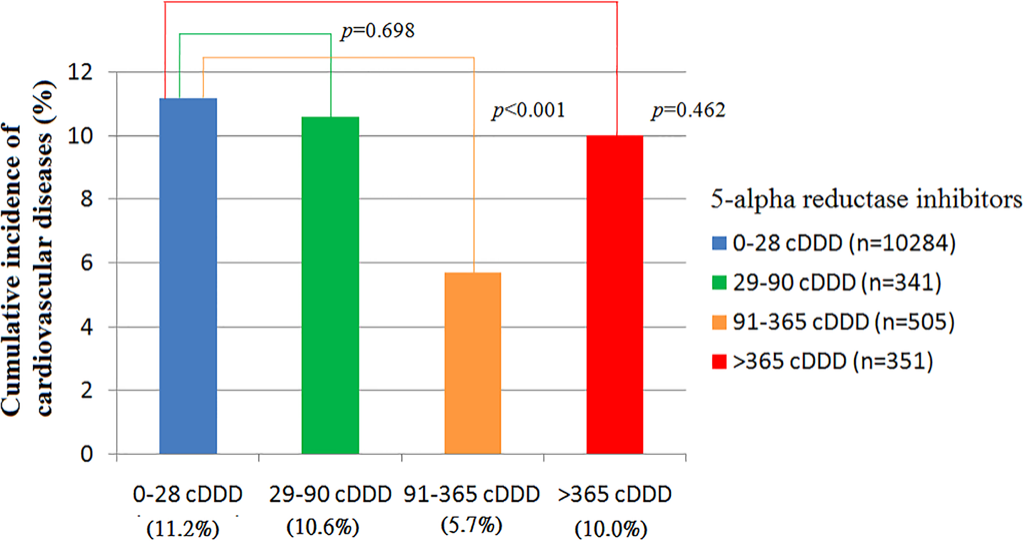
The 5-year Cumulative incidence of cardiovascular diseases in those receiving and not receiving 5-alpha reductase inhibitors.

**Fig 2:**
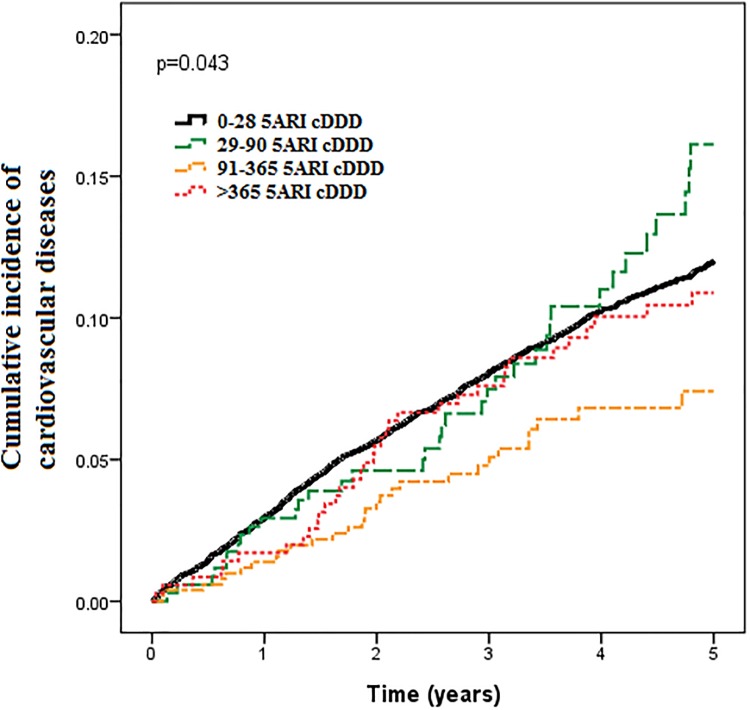
Kaplan-Meier failure curves of developing cardiovascular diseases in the patients receiving and not receiving 5-alpha reductase inhibitors.

At the end of the follow-up period, 1,255 patients had cardiovascular diseases, including 100 (8.4%) in the 5-alpha-reductase inhibitor therapy group and 1,155 (11.2%) in the control group (Table 2; P = 0.003). The 5-year cardiovascular event rates were 10.6%, 5.7% and 10.0% for the patients who received 5-alpha-reductase inhibitor therapy with 28 to 90, 91 to 365, and more than 365 cDDDs, respectively. There was a greater reduction in risk with 91 to 365 than 0 to 28 cDDDs of 5-alpha-reductase inhibitors (Fig. 1.; 5.7% vs. 11.2%, P<0.001). Fig. 2 shows the Kaplan-Meier failure curve of developing cardiovascular diseases in the patients by different cDDDs of 5-alpha-reductase inhibitors. The patients with 91 to 365 cDDDs had a significant failure rate of developing cardiovascular diseases compared to the other groups during the 5-year follow-up period (P = 0.043).

**Table 2:** The 5-year cumulative rate of cardiovascular diseases in those receiving and not receiving 5-alpha reductase inhibitor therapy.

Characteristics	*n*	Events (%)	*p*-value
5ARI status			0.003
0–28 cDDD	10284	1155(11.2)	
>28 cDDD	1197	100(8.4)	

Abbreviation: 5ARI, 5-alpha reductase inhibitor; cDDD: cumulative defined daily dose.

Adjusted HRs for cardiovascular disease between 5-alpha-reductase inhibitor use in different age groups are shown in Table 3 and 4. Table 3 shows the patients who were younger than 65 years. The cardiovascular disease HRs were not significantly different among the patients who received a cDDD of 5-alpha-reductase inhibitors of 0 to 28, 28 to 90, 91 to 365, and more than 365. However, there was a trend towards an increased risk with increasing Charlson Comorbidity Index Score. The adjusted HRs were 2.28 (95% CI, 1.72 to 3.03), 2.95 (95% CI, 2.00 to 4.35), and 3.21 (95% CI, 1.95 to 5.33) for the patients with Charlson Comorbidity Index Scores of 1, 2, and 3 or more, respectively (P<0.001).

**Table 3:** Multivariate adjusted hazard ratios (HR) of cardiovascular diseases (CVD) among younger (younger than 65 years old) patients receiving and not receiving 5-alpha reductase inhibitors from 2003 to 2008.

Variable	5-year CVD risk		
	Adjusted HR***	95% CI	*p*-value
Group			
0–28 cDDD	1		
29–90 cDDD	1.45	0.77–2.74	0.250
91–365 cDDD	0.38	0.12–1.19	0.095
>365 cDDD	1.01	0.45–2.28	0.977
Charlson Comorbidity Index Score			
0	1		
1	2.28	1.72–3.03	<0.001
2	2.95	2.00–4.35	<0.001
> = 3	3.21	1.93–5.33	<0.001
Socioeconomic status			
Low	1		
Moderate	0.86	0.64–1.15	0.307
High	0.70	0.52–0.95	0.022
Urbanization			
Urban	1		
Suburban	1.01	0.75–1.36	0.951
Rural	1.21	0.85–1.71	0.297
Geographic Region			
Northern	1		
Central	0.80	0.55–1.16	0.239
Southern	0.86	0.64–1.17	0.342
Eastern	0.78	0.32–1.94	0.597

CI, 95% confidence interval.

***Adjusted for socioeconomic status, urbanization, geographic region and Charlson Comorbidity Index Score.

**Table 4:** Multivariate adjusted hazard ratios (HR) of cardiovascular diseases (CVD) among older (65 years old or older) patients receiving and not receiving 5-alpha reductase inhibitors from 2003 to 2008.

Variable	5-year CVD risk		
	Adjusted HR***	95%CI	*p*-value
Group			
0–28 cDDD	1		
29–90 cDDD	1.14	0.77–1.68	0.518
91–365 cDDD	0.63	0.42–0.92	0.018
>365 cDDD	0.83	0.57–1.20	0.310
Charlson Comorbidity Index Score			
0	1		
1	1.36	1.17–1.58	<0.001
2	1.73	1.15–2.08	<0.001
> = 3	2.09	1.70–2.56	<0.001
Socioeconomic status			
Low	1		
Moderate	0.89	0.75–1.06	0.199
High	0.81	0.54–1.20	0.289
Urbanization			
Urban	1		
Suburban	1.13	0.96–1.33	0.140
Rural	1.09	0.89–1.33	0.408
Geographic Region			
Northern	1		
Central	1.02	0.85–1.22	0.842
Southern	1.03	0.88–1.19	0.748
Eastern	0.57	0.36–0.88	0.012

***Adjusted for socioeconomic status, urbanization, geographic region and Charlson Comorbidity Index Score.

Table 4 shows the patients who were older than 65 years. Compared with the controls, the 5-year cardiovascular disease HR was lower among the patients with 91 to 365 cDDDs of 5-alpha-reductase inhibitors (HR = 0.63; 95% CI, 0.42 to 0.92; P = 0.018), but not different the among patients with 28 to 90 and more than 365 cDDDs of 5-alpha-reductase inhibitors (HR = 1.14, 95% CI 0.77 to 1.68; P = 0.518, and HR = 0.83, 95% CI 0.57 to 1.20; P = 0.310, respectively). The patients who resided in eastern Taiwan also had a lower risk of cardiovascular diseases (HR = 0.57, 95% CI 0.36 to 0.88, P = 0.012). There was a trend towards an increased risk with increasing Charlson Comorbidity Index Score, with adjusted HRs of 1.36 (95% CI, 1.17 to 1.58), 1.73 (95% CI, 1.15 to 2.08), and 2.09 (95% CI, 1.70 to 2.56) for the patients with Charlson Comorbidity Index Scores of 1, 2, and 3 or more, respectively (P<0.001).

## Discussion

This study demonstrated that 5-alpha-reductase inhibitor therapy in BPH patients did not increase the risk of cardiovascular disease, even in the patients who were older than 65 years. Another interesting finding was that clinicians attempted to treat BPH patients with an older age and higher Charlson Comorbidity Index Score BPH with 5-alpha-reductase inhibitors. A reason for this may have been to avoid further surgery for BPH or due to the severity of symptoms in these patients, however this cannot be addressed by a retrospective study.

The relationship between the dosage of 5-alpha-reductase inhibitors and the risk of cardiovascular disease was a J-curve association (Fig. 1). A possible cause is that whether or not key risk factors are related linearly to the occurrence of cardiovascular events is controversial. This J-curve association was also observed in studies of cardiovascular risk between cholesterol or blood pressure thresholds [26–28].

The strengths of our study are that it was a nationwide population-based cross-sectional study, with nearly complete follow-up information with regards to health care institutes among the whole study population (99%), as well as the fact that the dataset was routinely monitored for diagnostic accuracy by the NHI Bureau of Taiwan. As there were considerable difference in age and comorbidities between the patients who did and did not receive 5-alpha-reductase inhibitor therapy, propensity score matching with age was applied to select the controls. The risk of cardiovascular diseases with 5-alpha-reductase inhibitor therapy did not increase in the Cox proportional hazard model using a matching method.

Andriole et al reported that the relative incidence of the composite category of cardiac failure was higher in the patients with dutasteride treatment than in the placebo group [16]. However, their study excluded older patients (75 years or older), those with a large prostate volume (>80 ml), and patients with severe International Prostate Symptom Scores (>25 or >20 or higher in the case of men taking alpha-blockers). Because of the increasing prevalence of BPH with increasing age, these excluded patients were those who were most likely to need 5-alpha-reductase inhibitor therapy for BPH, and this potentially limited the results. On the other hand, Fleshner et al investigated the safety and efficacy of dutasteride on prostate cancer progression in men aged 48–82 years who had a low-volume, Gleason score 5–6 prostate cancer and had chosen to be followed up with active surveillance, and the results were found to similar in the dutasteride group and controls [29].

Souverein et al conducted a population-based investigation and found that the current use of finasteride was not associated with hospital admission for ischemic heart disease [15]. Furthermore, Kaplan et al reported their results in older men (65 years or older) in whom there was no significant difference between the placebo and finasteride-treated patients in the incidence of cardiovascular adverse events [14]. These results are similar to our study and were more useful in clinical practice for BPH patients.

This study has several limitations. First, the diagnosis of cardiovascular disease and any other comorbid conditions were completely dependent on ICD-9-CM codes. Nonetheless, the NHI Bureau of Taiwan randomly reviews the charts and interviews patients to verify the accuracy of the diagnosis. Hospitals with outlier chargers or practice may undergo an audit, with subsequent heavy penalties for malpractice or discrepancies. Second, the severity of cardiovascular disease cannot be precisely extracted from ICD-9-CM codes, which prevented further subgroup analysis. Third, the database does not contain information on tobacco use, dietary habits, and body mass index, which may also be risk factors for cardiovascular diseases. Further studies linking administrative data and primary hospitalization information such as severity of cardiovascular disease and detailed risk factors are warranted. Nonetheless, given the magnitude and statistical significance of the observed effects in this study, these limitations are unlikely to have compromised the results.

## Conclusions

The results of the present study show that 5-alpha-reductase inhibitor therapy did not increase the risk of cardiovascular events in BPH patients in 5 years of follow-up. Furthermore, 5-alpha-reductase inhibitor therapy resulted in a lower 5-year cumulative rate for cardiovascular events in the BPH patients, especially in older patients (65 years or older) with 91 to 365 cDDDs of 5-alpha-reductase inhibitors. Further mechanistic research is needed.
